# Disrupted Functional Connectivity of Cornu Ammonis Subregions in Amnestic Mild Cognitive Impairment: A Longitudinal Resting-State fMRI Study

**DOI:** 10.3389/fnhum.2018.00413

**Published:** 2018-10-29

**Authors:** Hui Li, Xiuqin Jia, Zhigang Qi, Xiang Fan, Tian Ma, Ran Pang, Hong Ni, Chiang-shan R. Li, Jie Lu, Kuncheng Li

**Affiliations:** ^1^Department of Radiology, Xuanwu Hospital, Capital Medical University, Beijing, China; ^2^Beijing Key Laboratory of MRI and Brain Informatics, Beijing, China; ^3^Department of Radiology, Beijing Chaoyang Hospital, Capital Medical University, Beijing, China; ^4^Institute of Psychology, Chinese Academy of Sciences, Beijing, China; ^5^Department of Psychiatry, Yale School of Medicine, Yale University, New Haven, CT, United States; ^6^Department of Neuroscience, Yale School of Medicine, Yale University, New Haven, CT, United States; ^7^Beijing Huilongguan Hospital, Beijing, China

**Keywords:** cornu ammonis, hippocampus, amnestic mild cognitive impairment, functional connectivity, longitudinal study

## Abstract

**Background:** The cornu ammonis (CA), as part of the hippocampal formation, represents a primary target region of neural degeneration in amnestic mild cognitive impairment (aMCI). Previous studies have revealed subtle structural deficits of the CA subregions (CA1-CA3, bilateral) in aMCI; however, it is not clear how the network function is impacted by aMCI. The present study examined longitudinal changes in resting state functional connectivity (FC) of each CA subregion and how these changes relate to neuropsychological profiles in aMCI.

**Methods:** Twenty aMCI and 20 healthy control (HC) participants underwent longitudinal cognitive assessment and resting state functional MRI scans at baseline and 15 months afterward. Imaging data were processed with published routines in SPM8 and CONN software. Two-way analysis of covariance was performed with covariates of age, gender, education level, follow up interval, gray matter volume, mean FD, as well as global correlation (GCOR). Pearson’s correlation was conducted to evaluate the relationship between the longitudinal changes in CA subregional FC and neuropsychological performance in aMCI subjects.

**Results:** Resting state FC between the right CA1 and right middle temporal gyrus (MTG) as well as between the left CA2 and bilateral cuneal cortex (CC) were decreased in aMCI subjects as compared to HC. Longitudinal decrease in FC between the right CA1 and right MTG was correlated with reduced capacity of episodic memory in aMCI subjects.

**Conclusion:** The current findings suggest functional alterations in the CA subregions. CA1 connectivity with the middle temporal cortex may represent an important neural marker of memory dysfunction in aMCI.

## Introduction

Amnestic mild cognitive impairment (aMCI) precedes the onset of Alzheimer’s disease (AD) and comprises a syndrome of cognitive decline without notable interference with daily activities (Gauthier et al., 2006; Petersen, 2011). The hippocampus plays a critical role in the encoding, consolidation, storage, and retrieval of episodic memories (Ranganath et al., 2005; Okada and Okaichi, 2009; Langston et al., 2010). Abundant evidence has suggested that the hippocampus is one of the most specific and sensitive pathological sites of AD (Ulrich, 1985; Delacourte et al., 1999; Chetelat and Baron, 2003; Maruszak and Thuret, 2014).

The hippocampus is divided into fascia dentate (dentate gyrus or DG), subregions of the cornu ammonis (CA1-CA4), and subiculum (SUB). Using *in vivo* magnetic resonance imaging (MRI), architectonic maps of the CA1-CA3, the subicular complex, fascia dentata together with CA4, and the hippocampal-amygdaloid transition area have been generated (Amunts et al., 2005). Neuropathological studies revealed the progression of neurodegeneration throughout the hippocampus (Gomez-Isla et al., 1996; Schonheit and Zarski, 2004) and indicated that CA1 might be the first hippocampal subregion being affected by neurofibrillary changes and neuronal loss (Braak and Braak, 1991; Braak et al., 1993). Structural MRI studies demonstrated differential involvement of the hippocampal subfields in normal aging, MCI, AD, and semantic dementia (La Joie et al., 2010; de Flores et al., 2015). The patterns of volumetric reduction suggested most severe atrophy in the CA1, followed by SUB, CA2, and CA3/DG in MCI and AD patients, in association with cognitive deterioration (Apostolova et al., 2010; La Joie et al., 2013; Tang et al., 2015). These findings indicate that although structural deficits involve all of the hippocampal subregions, the onset time and severity may vary across them.

In contrast to structural MRI showing the anatomical characteristics, functional MRI measures brain activity by detecting changes in blood oxygenation level dependent (BOLD) signals. It has been proposed that functional alterations likely precede structural changes (Greicius et al., 2004; Fox and Raichle, 2007). Low-frequency fluctuation of the BOLD signals, as revealed by resting state functional MRI, provides a viable approach to assess the network functional integrity of structurally segregated brain regions. A previous resting state functional MRI work demonstrated dysconnectivity of the hippocampal subfields in aMCI subjects. Specifically, functional connectivity (FC) decreased between the SUB and posterior cingulate cortex (PCC), right ventromedial prefrontal cortex, as well as left superior frontal cortex in aMCI as compared to health control (HC) participants (de Flores et al., 2017). In addition, a longitudinal resting state functional MRI study demonstrated that aMCI subjects exhibited changes in FC of bilateral CA, SUB, and DG with the PCC, lateral temporal cortex, medial frontal gyrus, insula, and cerebellum over time (Bai et al., 2011).

However, the aforementioned studies did not distinguish the CA1-CA3 subregions. Previous studies have revealed that different CA subregions harbor distinct functions in learning and memory (Tsien et al., 1996; Nakashiba et al., 2008; Coras et al., 2014; Adnan et al., 2016; Sormaz et al., 2017; Shah et al., 2018). The CA1 has been considered as a mismatch detector of environmental inputs versus stored representations, as well as a contextual filter to associate temporally discontiguous stimuli (Wallenstein et al., 1998; Rolls and Kesner, 2006). In addition, along with CA2, CA1 contributes to the associative memory deficits seen in MCI subjects (Atienza et al., 2011). Investigated only very recently, the CA2 has been implicated in memory consolidation (Oliva et al., 2016), social memory (Hitti and Siegelbaum, 2014), and especially in spatial memory (Alexander et al., 2016). Lastly, the CA3 plays an important role in associating the stimuli-matched patterns with previous experiences (Nakazawa et al., 2002; Nakashiba et al., 2008). Thus, it would be important to examine how CA subregional resting state FC may be altered in aMCI, as compared to HC, cross-sectionally and longitudinally.

In the present study, we investigated whole-brain FC in individuals with aMCI and demographically matched HC, both at baseline and after 15 months of follow-up. On the basis of previous studies, we hypothesized that, in comparison with HC, aMCI subjects might demonstrate CA subregion-specific changes in FC with cortical and subcortical regions and these changes might aggravate as the illness progresses. Further, we explored how these functional brain changes relate to neuropsychological deficits in aMCI.

## Materials and Methods

### Participants

The study was conducted under a research protocol approved by the Ethics Committee of the Xuanwu Hospital. A written informed consent was obtained from all participants prior to the study.

Twenty aMCI subjects and 20 HC participants were recruited from the Xuanwu Hospital of Capital Medical University in Beijing. At baseline, all the participants underwent detailed evaluation of medical history, complete physical examination, MRI scan, and neuropsychological evaluation with Mini Mental State Examination (MMSE), Clinical Dementia Rating (CDR), Montreal Cognitive Assessment (MoCA), and Auditory Verbal Learning Test (AVLT). After 15 months, all the participants completed a follow-up MRI scan and neuropsychological evaluation. MRI parameters and neuropsychological tests at follow-up were identical to those undertaken at baseline.

The diagnosis of aMCI followed the criteria stipulated by the National Institute on Aging and the Alzheimer’s Association (Albert et al., 2011), which include: (a) complaint of cognitive change; (b) cognitive function impairment, especially in episodic memory; (c) ability to maintain independence in daily activities; (d) not demented; (e) CDR-SB (Sum of the Boxes) score = 0.5, with a score of at least 0.5 on the memory domain (Petersen et al., 2001); and (f) a score >1 of visual rating of medial temporal lobe atrophy on coronal Tl-weighted MRI (Scheltens et al., 1992).

The criteria for the HC were as follows: (a) no complaints of cognitive changes; (b) absence of abnormal findings in brain MRI; (c) no neurological deficiencies in physical examinations; (d) no current or previous diagnosis of any neurological or psychiatric disorders; and (e) CDR-SB score = 0.

Participants were excluded from the study if they had a previous diagnosis of stroke, head injury, alcohol use disorder, epilepsy, depression, Parkinson’s disease or other neurological or psychiatric illness, major medical illness, and severe visual or hearing loss. Additional exclusion criteria for both aMCI and HC participants included contraindications for MRI, such as the use of cardiac pacemakers and claustrophobia.

### Magnetic Resonance Imaging Procedures

The subjects were scanned using a 3-Tesla Trio scanner (Siemens, Erlangen, Germany). All the participants were asked to hold still, keep their eyes closed, and stay awake. Foam pads were employed to minimize head motion and headphones were used to reduce scanner noise. Resting state functional MRI images were acquired using an echo-planar imaging (EPI) sequence: repetition time (TR) = 2000 ms, echo time (TE) = 40 ms, flip angle (FA) = 90°, field of view (FOV) = 256 mm, 28 axial slices, slice thickness/gap = 4/1 mm, bandwidth = 2232 Hz/pixel, and number of repetitions = 239. The 3D T1-weighted anatomical images were acquired with a magnetization-prepared rapid gradient echo (MPRAGE) method with the following parameters: TR = 1900 ms, TE = 2.2 ms, inversion time (TI) = 900 ms, FA = 9°, bandwidth = 199 mm, matrix = 256 × 224, 176 sagittal slices, and slice thickness = 1 mm.

### MRI Data Preprocessing

Resting state functional MRI images were preprocessed using Statistical Parametric Mapping software SPM8^1^, and seed-to-voxel correlation analysis was carried out by the functional connectivity (CONN) toolbox v17c (Whitfield-Gabrieli and Nieto-Castanon, 2012). The first 10 images of each resting state functional MRI data set were discarded to reduce the initial fluctuation of MRI signals. Images of each individual subject were corrected for slice timing and realigned. Participants with head motion more than 1.5 mm in translation or more than 1.5° in rotation were excluded. The resulting images were normalized to the Montreal Neurological Institute (MNI) space and resampled to 2 mm × 2 mm × 2 mm. Subsequently, the functional images were smoothed with a 4-mm full width at half maximum (FWHM) isotropic Gaussian kernel. After preprocessing, images were then band-pass filtered to 0.008 ∼ 0.09 Hz to reduce the influence of noise. Further denoising involved regression of the six motion parameters and their first-order derivatives, regression of white matter and cerebrospinal fluid signals (Behzadi et al., 2007) and linear detrending.

### Gray Matter Volume Analysis

The six CA seeds (right- and left- hemispheric CA1, CA2, and CA3) and bilateral hippocampi were defined through the Anatomy Toolbox in SPM8 (Eickhoff et al., 2005). Structural data processing was performed using SPM8 including normalization into the standard MNI apace, segmentation, modulation, and smoothing with an 8-mm full-width at half-maximum Gaussian kernel. The gray matter volumes of bilateral hippocampi and the six CA-subfields were then extracted based on the smoothed modulated gray matter maps and the latter was included in data analyses as nuisance covariates to rule out the influence of volumetric differences on FC.

### Functional Connectivity Analysis

For individual subjects, a mean time series each for the six CA seeds was computed as the reference time course. The Pearson correlation coefficients between the seed and all other brain voxels were computed to generate correlation maps. For group analyses, the correlation coefficients were converted to z-value using Fisher’s r-to-z transformation to improve normality (Lowe et al., 1998).

### Statistical Analysis

Demographic data and neuropsychological measures were analyzed using SPSS 19. Student *t*-tests were applied to compare continuous variables, including age, years of education, and neuropsychological scores, and a chi-square test was applied to examine the differences in gender composition between groups.

In the random effects analyses of imaging data, we first determined the patterns of voxelwise CA subregional FC within each group, using one sample *t*-tests without covariates, and evaluated the results at cluster level *p* < 0.05 corrected for multiple comparisons using the family-wise error rate (FWE). We then employed a 2 (time: follow-up versus baseline) × 2 (group: aMCI versus HC) flexible factorial design, with four conditions – aMCI baseline (aMCI1), aMCI follow-up (aMCI2), HC baseline (HC1), and HC follow-up (HC2), to examine time and group main effects as well as time by group interaction effect. Results were thresholded at cluster level *p* < 0.05, FWE corrected. The covariates used in between-group analyses included age, gender, years of education, follow-up interval, gray matter volume, mean FD, as well as global correlation (GCOR) index. The GCOR index represented the average correlation coefficient between every pair of voxels across the entire brain, a measure of network centrality at each voxel computed for each subject during the denoising step in CONN (Saad et al., 2013). Brain regions were localized with software xjView^2^.

The group main effects were examined by [(HC1 + HC2) > (aMCI1 + aMCI2)] and [(aMCI1 + aMCI2) > (HC1 + HC2)] to determine decreased and increased FC in aMCI compared to HC participants for each CA subfield, respectively. The time main effects were evaluated by [(aMCI1 + HC1) > (aMCI2 + HC2)] and [(aMCI2 + HC2) > (aMCI1 + HC1)] to determine decreased and increased FC at follow-up compared to baseline, respectively. The time by group interaction effects were evaluated by [(aMCI2 > aMCI1) > (HC2 > HC1)] to identify increased FC of CA subregions in aMCI relative to HC and [(aMCI1 > aMCI2) > (HC1 > HC2)] to identify decreased FC of CA subregions in aMCI relative to HC.

*Post hoc* pairwise comparisons were conducted focusing on the CA subregions that showed significant main and interaction effects. Independent-samples *t*-tests were applied to compute the group differences of FC between HC and aMCI at baseline and follow-up separately. Decreased FC in aMCI group was determined by (HC1 > aMCI1) and (HC2 > aMCI2) while increased FC in aMCI group was determined by (aMCI1 > HC1) and (aMCI2 > HC2). Paired-samples *t*-tests were performed to explore the longitudinal FC changes within each group with (aMCI1 > aMCI2) and (HC1 > HC2) to identify decreased FC at follow-up and (aMCI2 > aMCI1) and (HC2 > HC1) to identify increased FC at follow-up, as compared to baseline.

### Correlation Analysis

Regions-of-interest (ROI) were defined based on the regions showing significant FC changes in CA subregions in aMCI compared to HC in the interaction effect analysis. For each subject the mean FC values across all voxels of each ROI was computed. Pearson correlation analysis was then conducted to evaluate the relationship between the longitudinal changes of the FC and longitudinal changes of neuropsychological data in aMCI subjects. Statistical significance was set at *p* < 0.05, Bonferroni corrected for multiple comparisons.

## Results

### Demographics and Neuropsychological Assessment

As shown in Table 1, aMCI and HC participants did not differ in gender, age, and education level while significant volumetric difference of the two groups in bilateral hippocampi were found. Compared to HC, aMCI group showed significant general cognitive decline as reflected by the scores in the MMSE (*p* < 0.001), MoCA (*p* < 0.001), and CDR (*p* < 0.001), as well as poorer memory performance as reflected by the AVLT immediate and delayed recall and recognition scores (*p* < 0.001 for all comparisons), both at baseline and follow-up. Compared to baseline, the subjects with aMCI showed worse cognitive performance in the MMSE (*p* = 0.017) and MoCA (*p* = 0.048) at follow-up, confirming more severe memory impairment as the disease progressed. Besides, two aMCI subjects developed mild AD while the others and all of the HC remained stable.

**Table 1:** Clinical characteristics of subjects with amnestic mild cognitive impairment (aMCI) and healthy control (HC).

	Baseline			Follow-up		
	HC	aMCI	*p*-value	HC	aMCI	*p*-value
Age (yrs)	67.25 ± 7.50	66.95 ± 9.65	0.91	68.67 ± 7.54	68.10 ± 9.67	0.84
Gender (m/f)	10/10	7/13	0.34	10/10	7/13	0.34
Education (yrs)	11.90 ± 3.82	9.65 ± 4.06	0.08	11.90 ± 3.82	9.65 ± 4.06	0.08
Volume of Lt. HP	0.562 ± 0.016	0.538 ± 0.026	0.002	0.560 ± 0.017	0.534 ± 0.027^†^	0.001
Volume of Rt. HP	0.538 ± 0.016	0.521 ± 0.027	0.020	0.535 ± 0.016^†^	0.516 ± 0.029^†^	0.013
CDR-SB	0 (20)	0.5 (20)	<0.001	0 (20)	0.5 (18)/1 (2)	<0.001
MMSE	28.60 ± 1.23	24.80 ± 3.70	<0.001	28.35 ± 1.46	23.45 ± 3.47^†^	<0.001
MoCA	26.40 ± 1.63	20.85 ± 4.70	<0.001	26.50 ± 1.87	19.50 ± 4.91^†^	<0.001
AVLT-immediate recall	8.88 ± 1.63	6.00 ± 1.45	<0.001	8.95 ± 1.25	6.08 ± 1.38	<0.001
AVLT-delayed recall	9.35 ± 3.54	4.55 ± 2.78	<0.001	9.25 ± 3.56	4.90 ± 3.02	<0.001
AVLT-recognition	11.25 ± 3.05	8.45 ± 3.36	<0.001	11.70 ± 2.22	7.75 ± 3.24	<0.001

*Values represent means ± SD; Lt., left; Rt., right; HP, hippocampus; CDR-SB, clinical dementia rate-sum of the boxes; MMSE, mini mental state examination; MoCA, Montreal Cognitive Assessment; AVLT, auditory verbal learning test; p-values were derived from Student t-tests comparing the two groups, except for “gender” where the p-value was obtained using the χ^2^-test; ^†^represents significant decline in follow-up vs. baseline.*

### CA Subregional FC: Within-Group Analysis

In HC group, both at baseline and follow-up, significant connectivities with the medial temporal lobe, including the hippocampus, parahippocampus, and amygdala, were identified for each of the six CA subregions (Supplementary Figures 1–6). Bilateral CA1 showed additional connectivities with bilateral superior/middle/inferior temporal gyri (Supplementary Figures 1, 2). In aMCI group, both at baseline and follow-up, FC patterns similar to HC group were revealed with relatively smaller cluster sizes (Supplementary Figures 1–6).

### CA Subregional FC: ANCOVA

The main effect of “group” revealed significantly decreased FC changes between right CA1 (CA1R) and right middle temporal gyrus (MTG), as well as left PCC in aMCI in contrast to HC. The main effect of “time” revealed decreased CA1R-FC with the right MTG and decreased CA2L-FC with bilateral cuneal cortex (CC) for follow-up in contrast to baseline.

**FIGURE 1:**
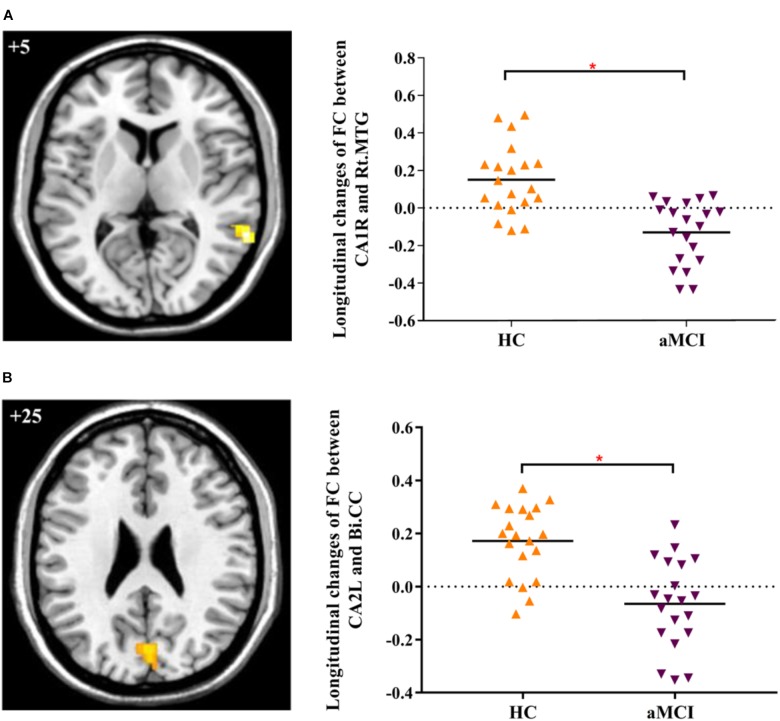
**(A)** Decreased FC between right CA1 and right MTG in aMCI subjects as compared to HC at follow-up vs. baseline (interaction effect); **(B)** Decreased FC between left CA2 and bilateral CC in aMCI subjects as compared to HC group at follow-up vs. baseline (interaction effect). Numbers in the figure indicate the *Z* coordinate in MNI space; FC, functional connectivity; CA1R, the right cornu ammonis 1; CA2L, the left cornu ammonis 1; Rt., right; Bi., bilateral; MTG, middle temporal gyrus; CC, cuneal cortex; ^∗^indicates cluster level *p* < 0.05, FWE corrected.

In addition, we explored the differences of longitudinal changes between aMCI and HC group in “group” by “time” interaction effect. The CA1R-FC showed greater decreases with the right MTG and CA2L-FC showed greater decreases with bilateral CC across time in aMCI in contrast to HC (Table 2 and Figures 1A,B).

**Table 2:** Longitudinal changes in functional connectivity to the cornu ammonis subregions in aMCI subjects as compared to HC.

Seed regions	Region	Cluster size (voxel)	MNI (x y z)			*F/T*-value
CA1R	HC vs. aMCI: [(HC1 + HC2) > (aMCI1 + aMCI2)]					
	Lt. PCC	190	−6	−46	36	12.67^∗^
	Rt. MTG	74	60	−48	8	16.01^∗^
	Baseline vs. Follow-up: [(aMCI1 + HC1) > (aMCI2 + HC2)]					
	Rt. MTG	114	64	−48	8	24.40^∗^
	[(aMCI1 > aMCI2) > (HC1 > HC2)]					
	Rt. MTG	108	64	−48	8	5.51
CA2L	Baseline vs. Follow-up: [(aMCI1 + HC1) > (aMCI2 + HC2)]					
	Bi. CC	66	−2	−76	24	18.95^∗^
	[(aMCI1 > aMCI2) > (HC1 > HC2)]					
	Bi. CC	125	0	−78	24	5.26

*Between-group results were thresholded at cluster-level p < 0.05 (FWE corrected); MNI, Montreal Neurological Institute; CA1R, the right cornu ammonis 1; CA2L, the left cornu ammonis 2; Lt., left; Rt., right; Bi., bilateral; PCC, posterior cingulate cortex; MTG, middle temporal gyrus; CC, cuneal cortex; ^∗^represents F-value.*

**FIGURE 2:**
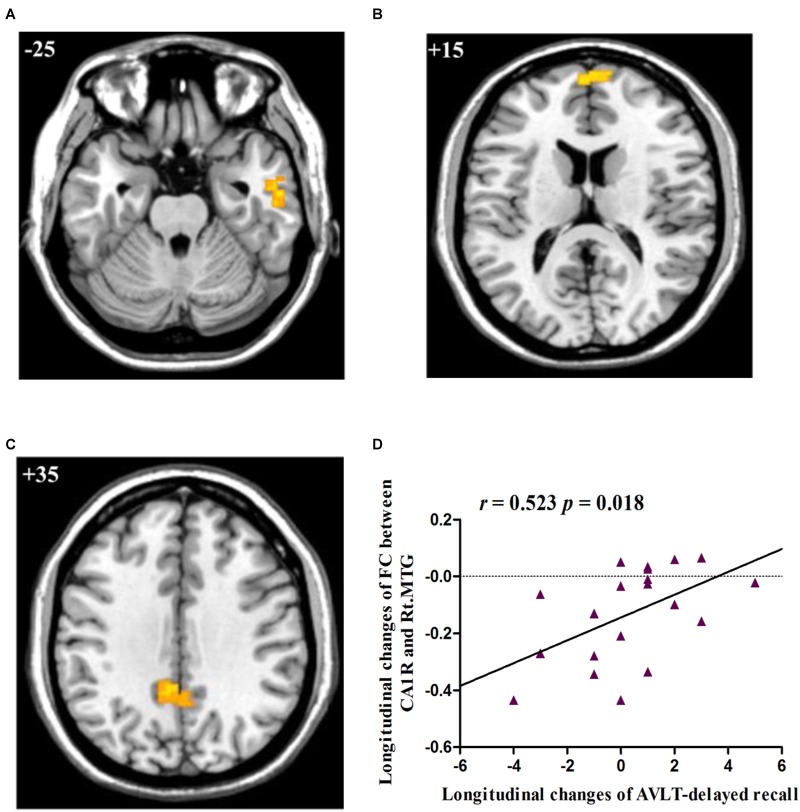
**(A)** Decreased FC between right CA1 and right MTG in aMCI subjects as compared to HC group at follow-up; **(B)** Decreased FC between right CA1 and right FP in aMCI subjects as compared to HC group at follow-up; **(C)** Decreased FC between right CA1 and left PCC in aMCI subjects as compared to HC group at follow-up; **(D)** Linear correlation of longitudinal changes in FC between right CA1 and right MTG and longitudinal changes of AVLT-delayed recall scores in aMCI subjects. Numbers in the figure indicate the *Z* coordinate in MNI space; FC, functional connectivity; CA1R, the right cornu ammonis 1; Lt., left; Rt., right; MTG, middle temporal gyrus; FP, frontal pole; PCC, posterior cingulate cortex; AVLT, Auditory Verbal Learning Test.

*Post hoc* pairwise comparisons showed that: 1: at baseline, no significant CA subregional FC changes were found in aMCI as compared to HC; 2: at follow-up, aMCI subjects demonstrated decreased CA1R-FC with right MTG (Figure 2A), right frontal pole (FP, Figure 2B), and left PCC (Figure 2C), as compared to HC; 3: no significant longitudinal FC changes were observed in aMCI or HC group.

### Correlation With Cognitive Measures

The two regions showing significant time by group interaction – the right MTG and bilateral CC – were defined as ROIs and the connectivity strength of each ROI was extracted for correlation analysis. The results showed that longitudinal changes (follow-up vs. baseline) in FC between the right CA1 and the right MTG were positively correlated with longitudinal changes (follow-up vs. baseline) in the capacity of episodic memory (*r* = 0.523, *p* = 0.018), as indexed by the delayed recall score of AVLT (Figure 2D).

## Discussion

We examined the differences in subjects with aMCI and healthy control (HC) participants in CA subregional (CA1-CA3) resting-state FC. Amnestic MCI subjects demonstrated decreased right CA1 (CA1R)-FC with the right MTG and left CA2 (CA2L)-FC with bilateral CC, in contrast to HC. Further, longitudinal changes of CA1R-FC with right MTG were associated with longitudinal changes of episodic memory across time. The findings suggest a potential hippocampal neural marker of dementia.

The CA1, an important region of both polysynaptic and monosynaptic pathways in the hippocampal mnemonic circuits, plays a crucial role in memory processing. Previous structural studies have suggested CA1 as the most vulnerable hippocampal subregions to be impacted in dementia (Apostolova et al., 2010; Tang et al., 2015). Morphological analyses revealed that decreases in gray matter volume and cortical thickness frequently occurred in the CA1 subfield in aMCI subjects (Yushkevich et al., 2015). A histopathological study demonstrated a significant association between CA1-specific atrophy and beta-amyloid deposition (Apostolova et al., 2015). Apostolova et al. (2015) also reported a significant association between changes in CA1 volume and neuronal count, which might contribute to memory dysfunction. Furthermore, atrophy of the CA1R was significantly associated with conversion to dementia (Apostolova et al., 2010) and appeared to be a reliable predictor of the development of AD in MCI subjects within a relatively short period of 3 years (Chow et al., 2015). Here, we demonstrated significantly decreased FC of CA1R over a 15-month follow-up period in aMCI subjects. The current results suggested crucial contribution of CA1 dysfunction to cognitive impairment in aMCI.

Consistent with a previous study, no significant CA subregional FC changes were found in aMCI subjects at baseline (de Flores et al., 2017). At follow-up, aMCI subjects as compared with HC subjects demonstrated significantly decreased FC between the CA1R and right MTG, right FP, as well as left PCC. Further, longitudinal changes in FC between the CA1R and right MTG were positively related to longitudinal changes of episodic memory as measured by delayed recall scores of AVLT, suggesting significant association between disrupted CA1R-FC and progressive cognitive alterations in aMCI. Functionally associated with verbal and visual semantic knowledge (Vandenberghe et al., 1996), the MTG supports verbal short-term memory (Raettig and Kotz, 2008). Previous studies reported that the MTG was one of the key brain regions of tau accumulation in aMCI and AD patients (Maass et al., 2017) and showed significant volume reduction during the disease process (Cha et al., 2013; Li K. et al., 2017). Further, abnormal FC between MTG and other brain regions, such as hypothalamus (Liu et al., 2018) and default mode network (Li M. et al., 2017), contributed to the cognitive impairment in aMCI and AD. In line with the aforementioned studies, decreases in the FC between CA1R and the right MTG may underline the role of the MTG in the clinical manifestation of aMCI and development of AD.

The CC, most known for its involvement in basic visual processing, is a key region of the occipito-parieto-frontal network in support of spatial information processing (Gomez et al., 2014). Active visual experiences induce visual cortex synaptic plasticity and this phenomenon can influence the following spatial episodes integration in hippocampus (Debanne, 1996; Tsanov and Manahan-Vaughan, 2008). Recent studies have implicated the CA2 in spatial information encoding and storage. Alexander et al. (2016) showed that the ability to modify the existing spatial representations by globally remapping the place fields was unique to neurons in the CA2. Moreover, CA2 neurons form a highly plastic disynaptic circuit to mediate key aspects of hippocampal-dependent spatial memory by integrating inputs from the entorhinal cortex and outputs to the CA1 (Chevaleyre and Siegelbaum, 2010). In the studies related with human hippocampal asymmetry, it has been suggested that, while the right hippocampus is related with an allocentric spatial representation concerning places, the left hippocampus significantly activates when using a sequential egocentric spatial representation, which likely associates with the spatiotemporal aspect of episodic memory (Igloi et al., 2010; Shipton et al., 2014; El-Gaby et al., 2015). In the present study, subjects with aMCI demonstrated decreased CA2L-FC with bilateral cuneal cortices. It is possible that the disrupted CA2L-FC may contribute to the spatial components of episodic memory impairment during the progression of cognitive impairment. The functional implications of this finding remain to be investigated in the future.

Several limitations need to be considered for this study. First, the mean follow-up intervals in aMCI and HC were group matched but there were inter-subject variations. Although this factor has been included as a nuisance covariate in data analyses, we cannot entirely rule out the influence of follow-up interval on the current results. Second, the sample size of the study was relatively small, and studies with a larger sample will be needed to replicate the current findings and to elucidate CA subregional functions and dysfunctions in aMCI. Third, without access to PET examination and cerebrospinal fluid analysis, the criteria used in the present study indicated an intermediate likelihood that the MCI syndrome is due to AD. The recently proposed A (beta-amyloid) – T (tau) – N (neuro-degeneration) system should be referred to in the future study (Jack et al., 2018).

In summary, we identified and dissociated FC alterations for CA subfields in subjects with aMCI across time. Specifically, functional dysconnectivity in the CA1R was prominently affected in aMCI, in association with cognitive decline, at 15-month follow-up. These new findings add to the literature of hippocampal dysfunction and may facilitate future research of neural markers of cognitive decline in aMCI and AD.

## Author Contributions

HL and XJ carried out data collection and analysis and wrote the manuscript. ZQ and JL helped with data interpretation. XF, TM, RP, and HN carried out data collection. C-sL contributed to the conceptualization of the study and revision of the manuscript. KL contributed to the conceptualization and design of the study and revised the manuscript.

## Conflict of Interest Statement

The authors declare that the research was conducted in the absence of any commercial or financial relationships that could be construed as a potential conflict of interest.The reviewer HG and handling Editor declared their shared affiliation.

## Footnotes

1 https://www.fil.ion.ucl.ac.uk/spm

2 http://www.alivelearn.net/xjview
