# Supplementary material for: Disrupted Functional Connectivity of Cornu Ammonis Subregions in Amnestic Mild Cognitive Impairment: A Longitudinal Resting-State fMRI Study
Source: Front Hum Neurosci. 2018 Oct 29;12:413. doi: 10.3389/fnhum.2018.00413 (PMC6216144; doi:10.3389/fnhum.2018.00413)

Supplementary materials

Article Title: Disrupted Functional Connectivity of Cornu Ammonis Subregions in Amnestic Mild Cognitive Impairment: A Longitudinal Resting-State fMRI Study

Figure 1. Whole brain functional connectivity of left CA1-FC within HC and aMCI groups for baseline (T1) and follow-up (T2) respectively. Numbers in the figure indicate the *Z* coordinate in Montreal Neurological Institute; colorbar indicates *t*-value.

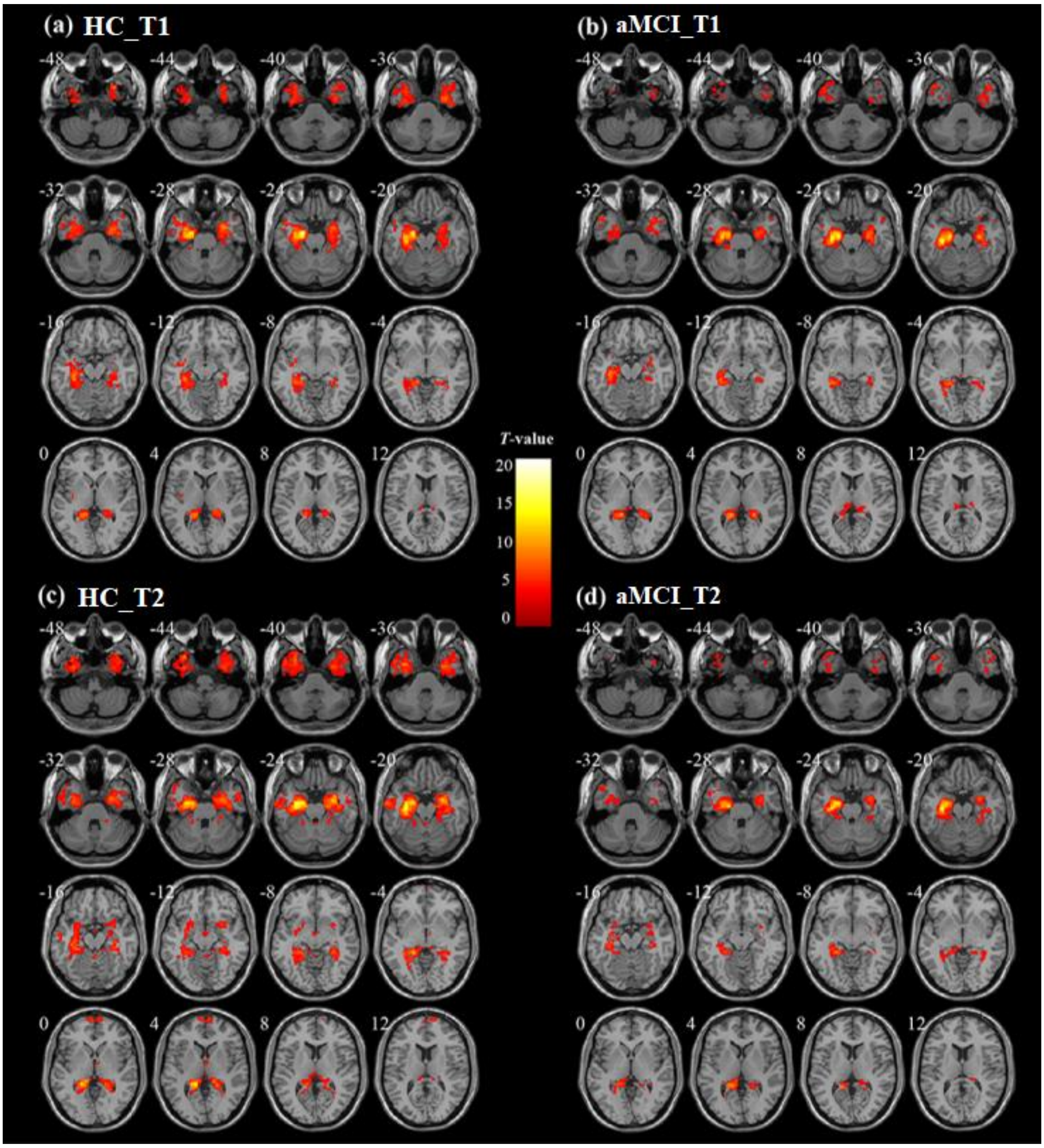

Figure 2. Whole brain functional connectivity of right CA1-FC within HC and aMCI groups for baseline (T1) and follow-up (T2) respectively. Numbers in the figure indicate the *Z* coordinate in Montreal Neurological Institute; colorbar indicates *t*-value.

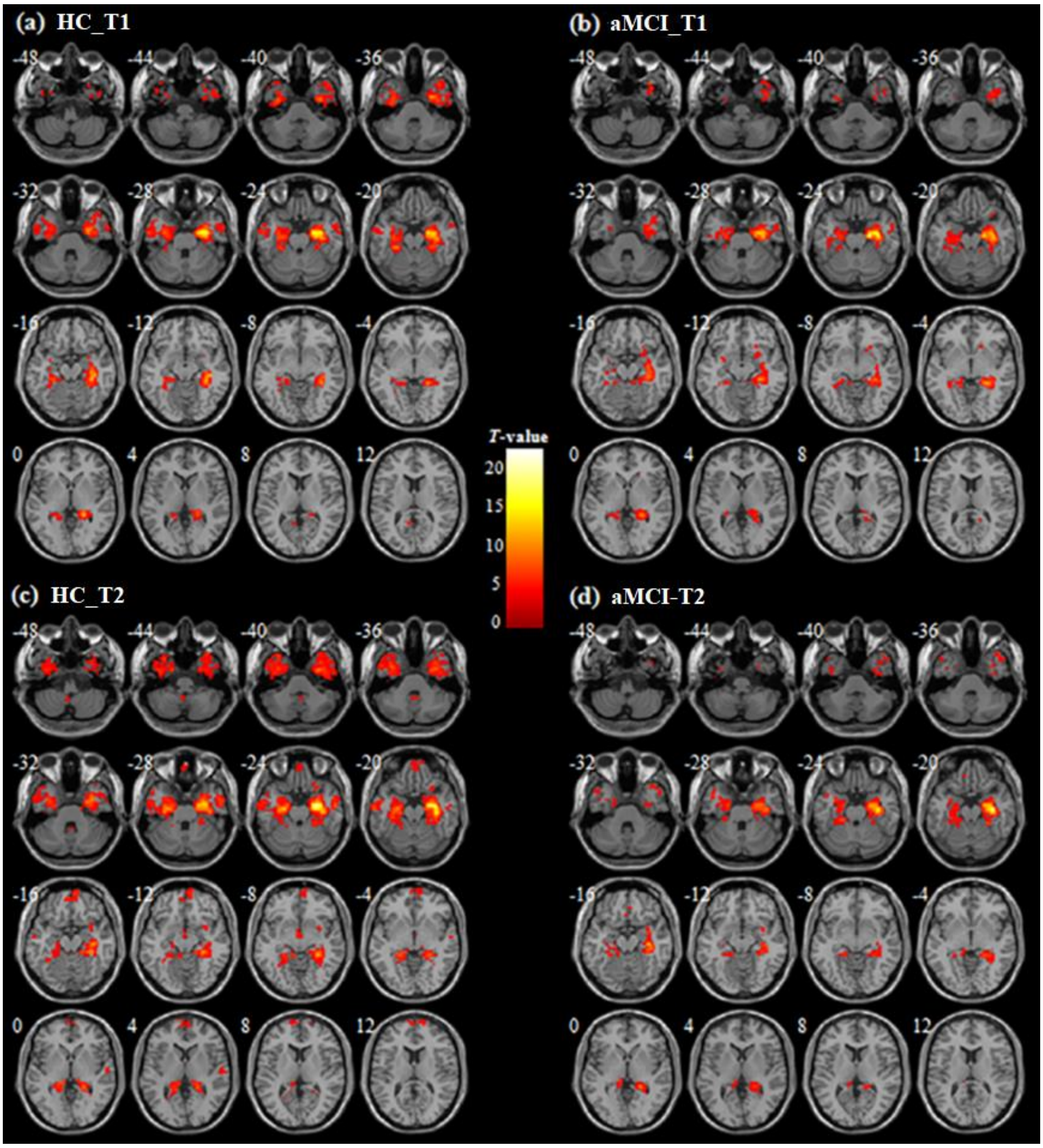

Figure 3. Whole brain functional connectivity of left CA2-FC within HC and aMCI groups for baseline (T1) and follow-up (T2) respectively. Numbers in the figure indicate the *Z* coordinate in Montreal Neurological Institute; colorbar indicates *t*-value.

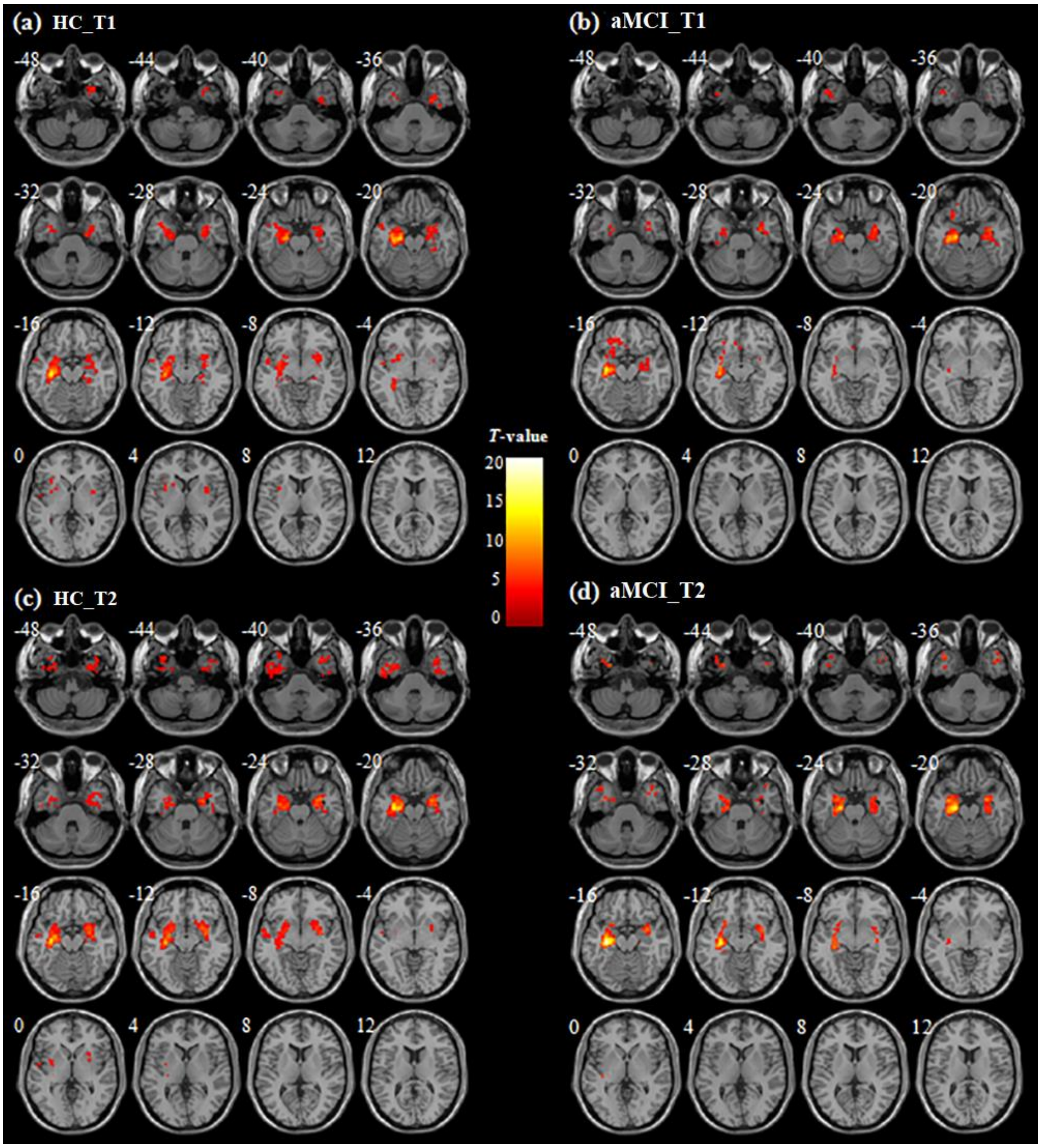

Figure 4. Whole brain functional connectivity of right CA2-FC within HC and aMCI groups for baseline (T1) and follow-up (T2) respectively. Numbers in the figure indicate the Z coordinate in Montreal Neurological Institute; colorbar indicates *t*-value.

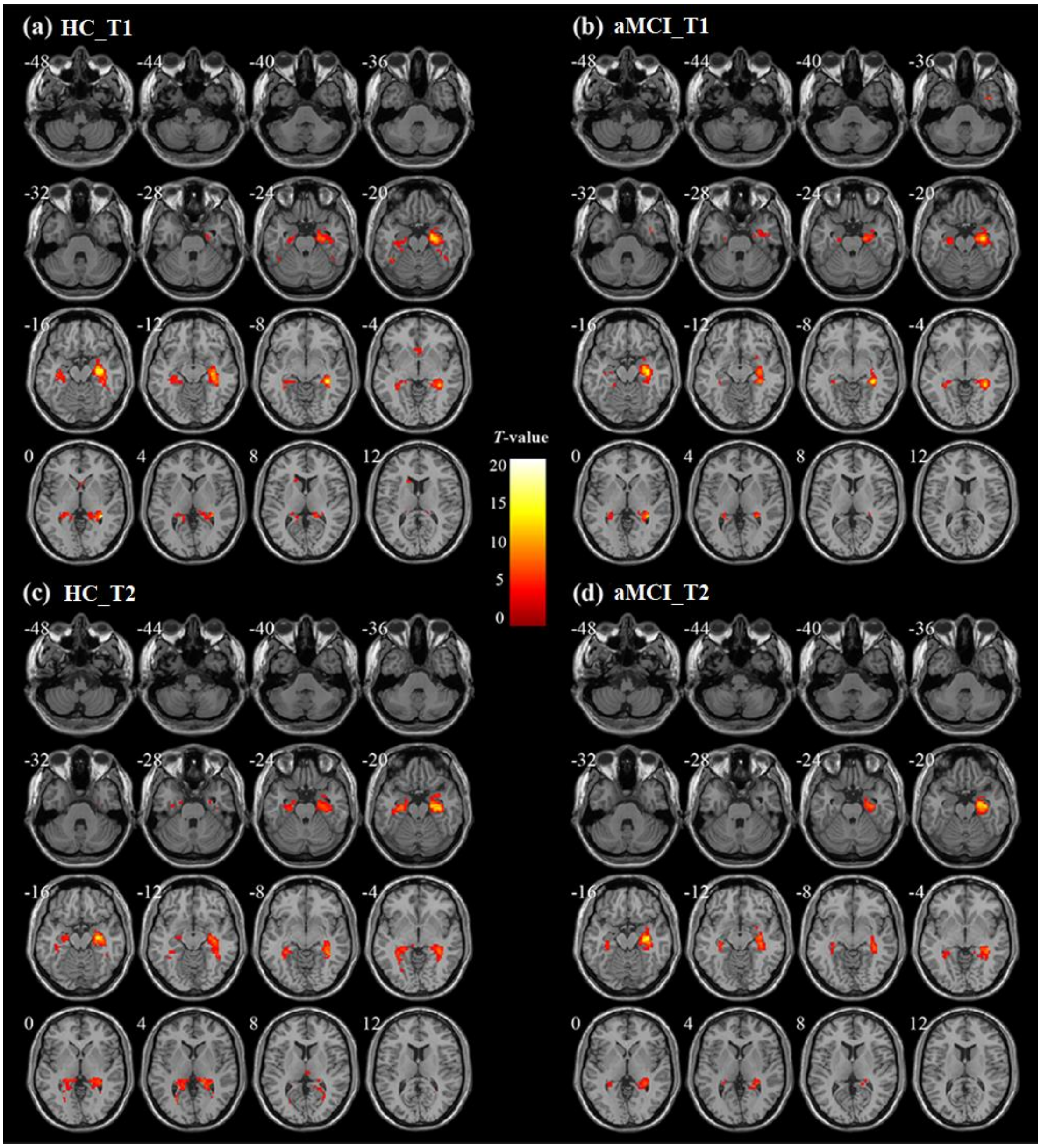

Figure 5. Whole brain functional connectivity of left CA3-FC within HC and aMCI groups for baseline (T1) and follow-up (T2) respectively. Numbers in the figure indicate the *Z* coordinate in Montreal Neurological Institute; colorbar indicates *t*-value.

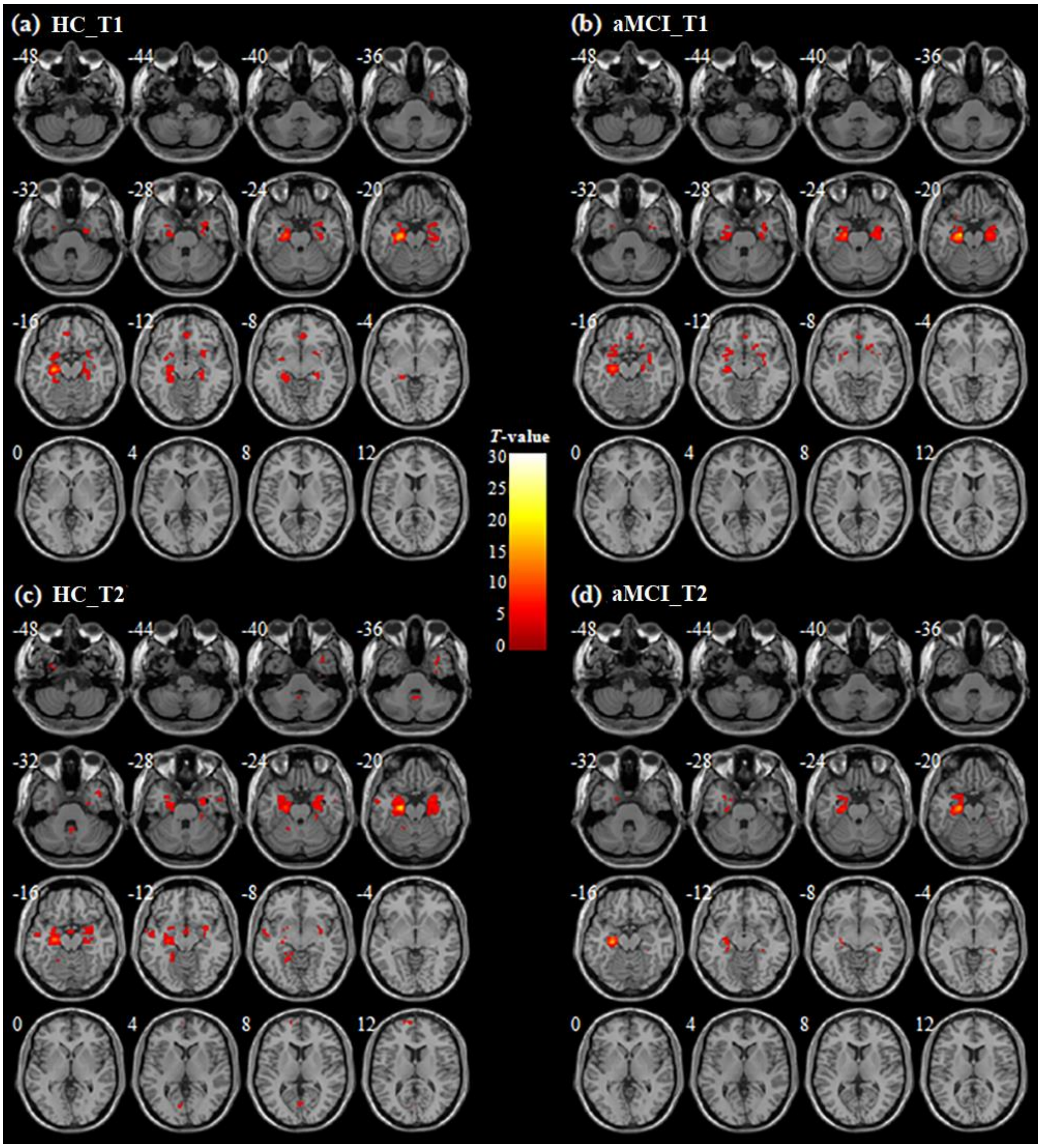

Figure 6. Whole brain functional connectivity of right CA3-FC within HC and aMCI groups for baseline (T1) and follow-up (T2) respectively. Numbers in the figure indicate the *Z* coordinate in Montreal Neurological Institute; colorbar indicates *t*-value.

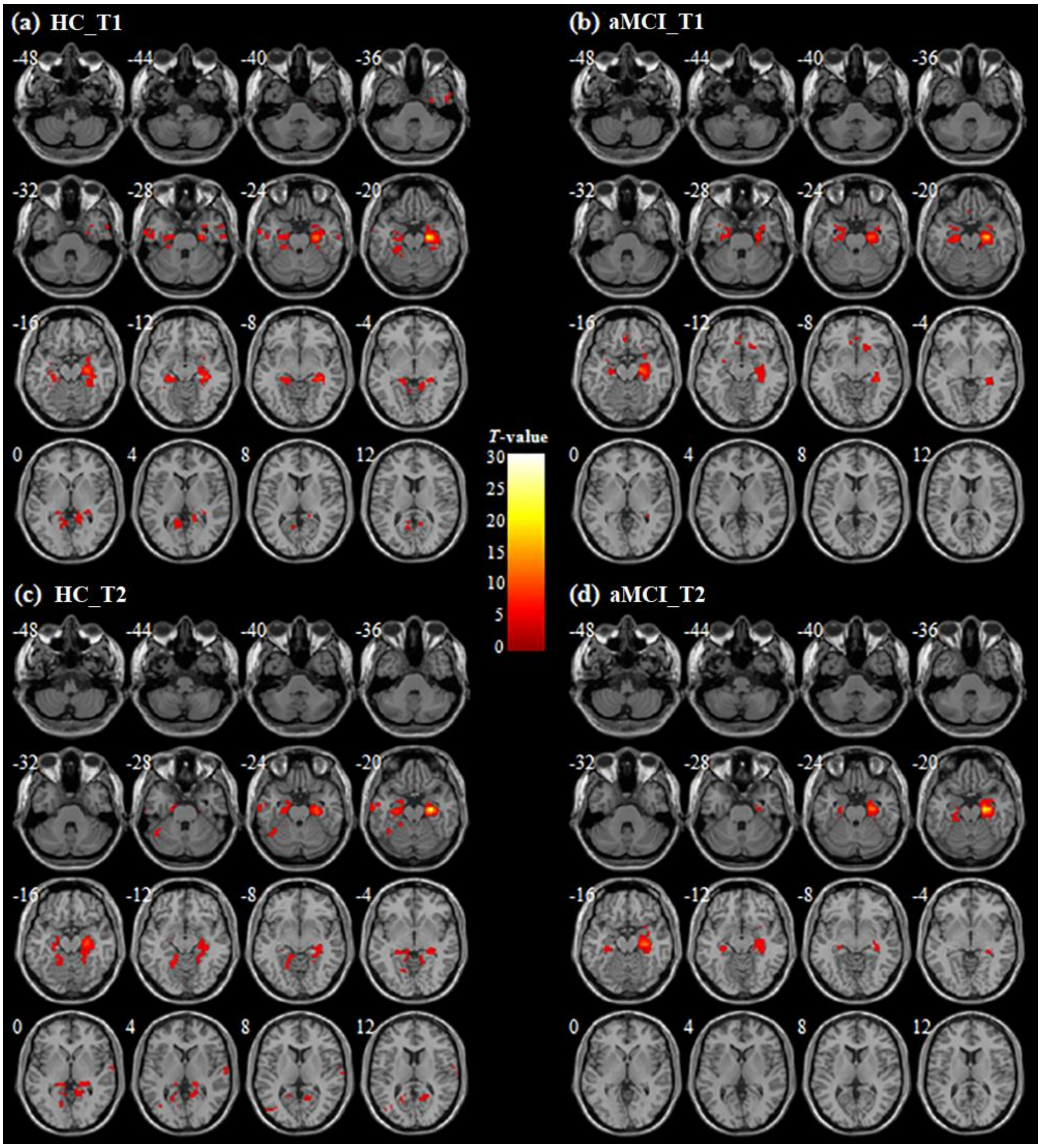

Supplement: Supplementary file 1 [file Data_Sheet_1.pdf]
